# Wie steht es um die Gesundheit pflegender erwerbstätiger Personen? Analyse einer Erwerbstätigenbefragung mittels Propensity Score Matching

**DOI:** 10.1007/s00391-024-02387-0

**Published:** 2025-01-06

**Authors:** Jan Mathis Elling, Christian Hetzel, Sarah Hampel, Adelheid von Spee, Greta Ollertz

**Affiliations:** 1 iqpr – Institut für Qualitätssicherung in Prävention und Rehabilitation GmbH, an der Deutschen Sporthochschule Köln, Eupener Str. 70, 50933 Köln, Deutschland; 2 Kuratorium Deutsche Altershilfe gGmbH (KDA), Köln, Deutschland

**Keywords:** Informelle Pflege, Häusliche Pflege, Angehörigenpflege, Matching, Vereinbarkeit, Nursing, Home care, Care by relatives, Matching, Work-care balance

## Abstract

**Hintergrund:**

Der Großteil der pflegebedürften Menschen in Deutschland wird zu Hause von Angehörigen oder anderen Bezugspersonen gepflegt. Viele informell Pflegende sind zudem erwerbstätig und stehen somit vor der Herausforderung, die Anforderungen beider Lebensbereiche zu vereinbaren. Dabei besteht die Gefahr, dass die eigene Gesundheit der Pflegenden leidet.

**Fragestellungen:**

(1) Inwiefern steht eine Pflegeverantwortung im privaten Umfeld bei Erwerbstätigen mit gesundheitlichen Beschwerden im Zusammenhang? (2) Innerhalb der Gruppe der pflegenden erwerbstätigen Personen, inwiefern stehen der Umfang von informeller Pflege *und* Erwerbsarbeit mit gesundheitlichen Beschwerden im Zusammenhang?

**Material und Methoden:**

Die Datenbasis dieser Sekundärdatenanalyse ist die BIBB/BAuA-Erwerbstätigenbefragung 2018. Um systematische Strukturunterschiede zwischen Pflegenden und Nichtpflegenden in den Analysen zu berücksichtigen, wurde das Propensity Score Matching angewendet.

**Ergebnisse und Diskussion:**

Pflegende Erwerbstätige haben häufiger psychosomatische und körperliche Beschwerden als Erwerbstätige ohne Pflegeverantwortung. Erwerbsarbeitszeit und Pflegeumfang stehen in Beziehung zueinander, und ein hoher Umfang in beiden Lebensbereichen ist mit schlechterer Gesundheit assoziiert.

**Schlussfolgerung:**

Personen, die neben ihrer Berufstätigkeit Pflegeaufgaben übernehmen, sind häufig gesundheitlich belastet. Daher sind gezielte Interventionen zur Gesundheitsförderung notwendig, um diese Gruppe zu unterstützen und ihre Gesundheit zu verbessern.

**Zusatzmaterial online:**

Zusätzliche Informationen sind in der Online-Version dieses Artikels (10.1007/s00391-024-02387-0) enthalten.

## Abstract (en)

**Background:**

The majority of people in need of care in Germany are cared for at home by relatives or other close individuals. Many informal caregivers are also in paid employment and face the challenge of juggling the demands of both areas of life. This can adversely affect the caregiver’s own health.

**Objectives:**

(1) To what extent is informal caregiving related to health complaints among people in paid employment? (2) Within the group of working caregivers, how are the amounts of informal care and work related to health complaints?

**Material and methods:**

This secondary data analysis is based on the BIBB/BAuA employment survey 2018. To account for systematic differences between caregivers and non-caregivers in the analyses, propensity score matching was applied.

**Results:**

Working caregivers more frequently experience psychosomatic and physical complaints compared to people in paid employment without caregiving responsibilities. The working hours and the extent of caregiving are interrelated and a high burden in both areas of life is associated with poorer health.

**Conclusion:**

People who provide informal care in addition to their work are often burdened with health problems. Targeted health promotion interventions are therefore necessary to support this vulnerable group and improve their health.

## Hintergrund und Fragestellung

Der demografische Wandel in Deutschland wird zu einem deutlichen Anstieg an pflegebedürftigen Personen führen. Prognosen zufolge wird allein durch die zunehmende Alterung der Gesellschaft mit einem Anstieg der Pflegebedürftigen bis 2055 um 37 % gerechnet [1]. Während im Jahr 2020 auf eine potenziell pflegebedürftige Person (im Alter von 80 Jahren oder älter) in Deutschland noch 4 potenzielle Pflegepersonen (im Alter zwischen 45 und 64 Jahren) kamen, werden es laut Prognosen im Jahr 2050 nur noch 2 potenzielle Pflegepersonen sein [2]. Diese Entwicklungen führen zu einer wachsenden Diskrepanz zwischen Pflegebedarf und Pflegeverfügbarkeit, wobei die formellen und informellen Pflegesysteme – und die Menschen in diesen Systemen – schon heute unter Druck stehen [3].

### Informelle Pflege

Der Großteil der pflegebedürften Menschen wird zu Hause gepflegt [4]. Informelle Pflege bezieht sich in der vorliegenden Arbeit auf Angehörige, Freunde oder Nachbarn, die nicht erwerbsmäßig Sorge- und Pflegeleistungen übernehmen. Dabei muss nicht zwingend ein Pflegegrad vorliegen. Die Pflegeleistungen können vielfältig sein und von alltäglichen Unterstützungsleistungen bis hin zur umfassenden Betreuung reichen. Auch nach dem Umzug der pflegebedürftigen Person in eine stationäre Einrichtung setzt sich die Begleitung durch informelle Pflegende oft fort. Unter den abhängig Beschäftigten in Deutschland pflegen 10 % der Frauen und 8 % der Männer jemanden im privaten Umfeld [5]. In der Altersgruppe der 50- bis 59-Jährigen sind es sogar 15 % der Frauen und 12 % der Männer [5].

### Vereinbarkeit von Beruf und Pflege

Deutlich wird: Viele informell Pflegende vereinbaren die Anforderungen ihrer Pflegeverantwortung mit beruflichen Verpflichtungen. Diese Personen, die sowohl einer Erwerbsarbeit nachgehen als auch im privaten Umfeld pflegen, stehen im Fokus der vorliegenden Studie. Die „Sandwich-Position“, in der sich diese Personen befinden, kann eine erhebliche Belastung darstellen und negative Folgen für die Gesundheit haben [6].

Die Forschung zur Gesundheit pflegender Angehöriger i. Allg. zeigt, dass es sich im Vergleich zu nichtpflegenden Personen um eine vulnerable Gruppe, die durch ein geringeres Wohlbefinden und eine schlechtere Gesundheit gekennzeichnet ist, handelt [7–11]. Gleichzeitig kann die Übernahme von Pflegeverantwortung auch positive und sinnstiftende Aspekte mit sich bringen, wie etwa das Gefühl, nützlich zu sein [10, 12, 13]. Die parallele Ausübung einer Erwerbsarbeit kann für informell Pflegende sowohl eine Belastung als auch eine Ressource darstellen. Für die Gesundheit ist zumeist entscheidend, wie gut die unterschiedlichen Rollen und Anforderungen *vereinbart* werden können [14, 15].

### Forschungsfragen

Die vorliegende Studie erweitert die vorhandene Literatur um Analysen zu gesundheitlichen Beschwerden bei pflegenden erwerbstätigen Personen und insbesondere zur Interaktion von Pflegeumfang und Arbeitszeit auf die gesundheitlichen Beschwerden. Die Forschungsfragen dieser Studie lauten:

1. Inwiefern steht eine Pflegeverantwortung im privaten Umfeld bei Erwerbstätigen mit gesundheitlichen Beschwerden im Zusammenhang?
2. Innerhalb der Gruppe der pflegenden erwerbstätigen Personen, inwiefern stehen der Umfang von informeller Pflege *und* Erwerbsarbeit mit gesundheitlichen Beschwerden im Zusammenhang?

## Methode

### Studiendesign

Die vorliegende Sekundärdatenanalyse basiert auf Daten der BIBB/BAuA-Erwerbstätigenbefragung 2018 [16]. Die Befragung mit dem Themenschwerpunkt Berufstätigkeit wurde vom Bundesinstitut für Berufsbildung (BIBB) und der Bundesanstalt für Arbeitsschutz und Arbeitsmedizin (BAuA) konzipiert und umgesetzt. Die Datenerhebung erfolgte mittels computergestützter telefonischer Interviews durch Kantar Public (ehemalige Abteilung der Kantar-Gruppe). Alle Daten sind dementsprechend selbstberichtet. Grundgesamtheit der Erwerbstätigenbefragung sind erwerbstätige Personen ab 15 Jahren mit einer bezahlten Tätigkeit von mindestens 10 h/Woche in Deutschland.

### Variablen

Eine ausführliche Darstellung des Erhebungsinstruments ist anderswo zu finden [17]. Die Frage zu den gesundheitlichen Beschwerden lautete: „Sagen Sie mir bitte, ob die folgenden gesundheitlichen Beschwerden bei Ihnen in den letzten 12 Monaten während der Arbeit bzw. an Arbeitstagen aufgetreten sind. Uns interessieren die Beschwerden, die häufig vorkamen“ und wurde auf einer dichotomen Antwortskala erfasst. Die gesundheitlichen Beschwerden wurden anschließend unterteilt in psychosomatische Beschwerden und Muskel-Skelett-Beschwerden [18]. Die Kategorie der psychosomatischen Beschwerden umfasst Beschwerden, die häufig durch psychische Belastungen verursacht oder verstärkt werden. Da von der Symptomatik grundsätzlich nicht auf die Ursachen geschlossen werden kann, ist darauf hinzuweisen, dass die Beschwerden auch primär körperliche Ursachen haben können.

### Statistische Analysen

Die Stichprobenmerkmale sind, nach Pflegeverantwortung differenziert, deskriptiv dargestellt. Für kontinuierliche und dichotome Variablen sind zudem die standardisierten Mittelwertdifferenzen und das dazugehörige 95 %-Konfidenzintervall angegeben. Zusätzlich werden im Text *p*-Werte berichtet, welche mittels *t*-Test bzw. Chi-Quadrat-Test berechnet wurden.

Um systematische Strukturunterschiede zwischen Pflegenden und Nichtpflegenden in den Analysen zu berücksichtigen, wurde das Propensity Score Matching angewendet. Im Propensity Score Matching wurden die Kovariaten Alter, Geschlecht, Familienstand, Kinder, Kinder unter 18 Jahre im Haushalt, Bildung, Branche, Jahre der Betriebszugehörigkeit, Arbeitslosigkeit und Nebentätigkeit berücksichtigt. Dabei kam die Methode des Nearest Neighbor Matching ohne Zurücklegen zum Einsatz. Die Propensity Scores wurden mit einem generalisierten linearen Modell geschätzt. Ein Verhältnis von 1:5 (Pflegende zu Nichtpflegenden) wurde gewählt, um die Anzahl der einbezogenen Daten der nichtpflegenden Personen zu maximieren und zugleich eine hohe Übereinstimmung zwischen den pflegenden und den nichtpflegenden Personen sicherzustellen. Das Einkommen und die Betriebsgröße wurden aufgrund der hohen Anzahl fehlender Werte für diese beiden Variablen nicht als Kovariaten in das Matching einbezogen.

Alle statistischen Analysen wurden in der Programmiersprache R (Version 4.4.0; [19]) unter Verwendung der Pakete MatchIt (Version 4.5.5; [20]) und cobalt (Version 4.5.5; [21]) durchgeführt.

## Ergebnisse

### Stichprobe

Die Grundgesamt der Erwerbstätigenbefragung umfasst 20.012 Personen. Hiervon haben 1564 Personen angegeben, eine Pflegeverantwortung im privaten Umfeld zu übernehmen. Keine Pflegeverantwortung trugen 18.417 Personen; 31 Personen verweigerten eine Aussage dazu. Tab. 1 zeigt die Deskription der Soziodemografie in der Gruppe der pflegenden Erwerbstätigen und der Gruppe der Erwerbstätigen ohne Pflegeverantwortung. Alle Gruppenunterschiede sind statistisch signifikant bei *p* < 0,001. Auffällig sind das höhere Durchschnittsalter (Differenz 4,1 Jahre) und der höhere Frauenanteil (62 %) in der Gruppe der Pflegenden. Während der Anteil der Personen mit Kindern unter den Pflegenden höher ist als unter den Nichtpflegenden, ist der Anteil der Personen mit Kindern unter 18 Jahren im Haushalt bei den Pflegenden niedriger.

**Tab. 1:** Deskription der Stichprobe, differenziert nach Pflegeverantwortung

Merkmal	Pflegende Erwerbstätige^1^ *n* = 1564	Nichtpflegende Erwerbstätige^1^ *n* = 18.417	SMD^2^	95 %-KI^3^
*Alter in Jahren*	50,9 (9,6)	46,9 (11,4)	0,39	0,33; 0,44
*Geschlecht*			0,27	0,22; 0,32
Männlich	38 %	51 %	–	–
Weiblich	62 %	49 %	–	–
*Familienstand*				
Verheiratet	63 %	55 %	–	–
Ledig	21 %	30 %	–	–
Geschieden	13 %	13 %	–	–
Verwitwet	3 %	3 %	–	–
*Kinder*			0,23	0,18; 0,28
Ja	77 %	67 %	–	–
Nein	23 %	33 %	–	–
*Kinder unter 18 Jahre im Haushalt*			0,11	0,06; 0,16
Ja	27 %	33 %	–	–
Nein	73 %	67 %	–	–
*Bildung*				
Ohne Berufsabschluss	5 %	5 %	–	–
Berufsausbildung	54 %	48 %	–	–
Aufstiegsfortbildung	9 %	8 %	–	–
Hochschulabschluss	32 %	39 %	–	–

^1^Mittelwert (SD)/%

^2^SMD: standardisierte Mittelwertdifferenzen

^3^KI: Konfidenzintervall

### Matching

**Abb. 1:**
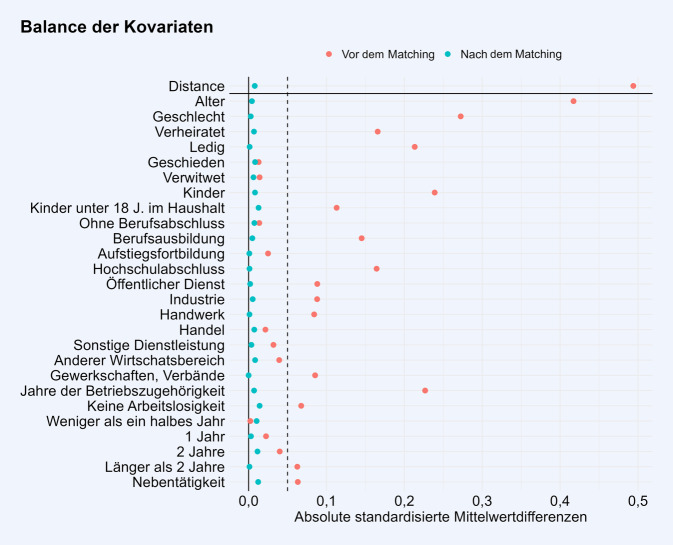
Absolute standardisierte Mittelwertdifferenzen der Propensity Scores („distance“) und Kovariaten vor (*orangefarbene Punkte*) und nach (*türkise Punkte*) dem Matching

Der oberste Wert („distance“) in Abb. 1 zeigt die standardisierte Mittelwertdifferenz in den Propensity Scores zwischen der Gruppe der Pflegenden und der Gruppe ohne Pflegeverantwortung. Vor dem Matching ist ein moderater Gruppenunterschied sichtbar. Nach dem Matching unterscheiden sich die Propensity Scores in den beiden Gruppen kaum mehr voneinander.

Abb. 1 zeigt des Weiteren die standardisierten Mittelwertdifferenzen aller in das Matching einbezogenen Kovariaten vor und nach dem Matching. Nach dem Matching liegen alle absoluten standardisierten Mittelwertdifferenzen unter 0,05 (*gestrichelte senkrechte Linie* in Abb. 1). Eine standardisierte Mittelwertdifferenz < 0,1 wird gemeinhin als vernachlässigbare Differenz im Mittelwert einer kontinuierlichen Kovariate oder in der Prävalenz einer dichotomen Kovariate angesehen [22]. Die durchgezogene senkrechte Linie kennzeichnet eine Mittelwertsdifferenz von 0 und somit gleiche Mittelwerte oder Prävalenzen in beiden Gruppen. Es ist sichtbar, dass eine Balance der Kovariaten vorliegt und das Matching somit die Gruppenunterschiede in den betrachteten Kovariaten erfolgreich reduziert bzw. beseitigt hat. Eine tabellarische Deskription der Kovariaten vor dem Matching ist im Zusatzmaterial online: Appendix 1 zu finden. Durch fehlende Werte in den Kovariaten wurde die Gruppe der Pflegenden auf 1505 Personen reduziert. Demgegenüber stehen 7525 Beschäftigte ohne Pflegeverantwortung, die durch das Matching zugeordnet wurden.

### Gesundheitliche Beschwerden

**Abb. 2:**
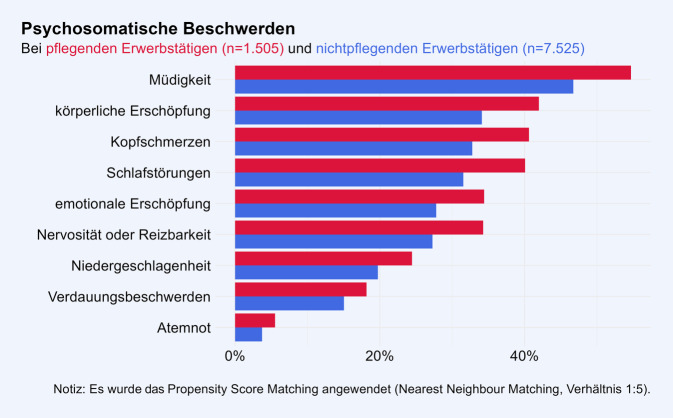
Psychosomatische Beschwerden in der Gruppe der pflegenden Erwerbstätigen und der Vergleichsgruppe ohne Pflegeverantwortung

**Abb. 3:**
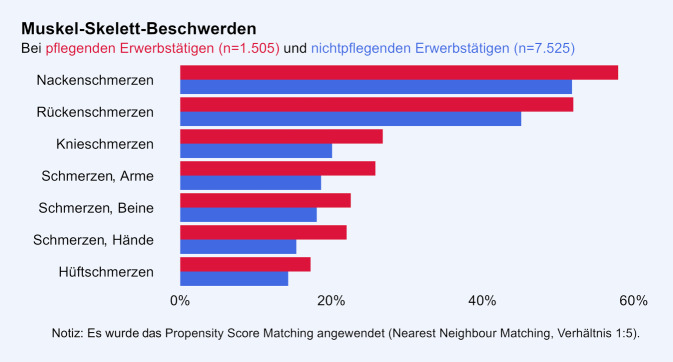
Muskel-Skelett-Beschwerden in der Gruppe der pflegenden Erwerbstätigen und der Vergleichsgruppe ohne Pflegeverantwortung

Die Gruppe der Pflegenden hat häufiger gesundheitliche Beschwerden, verglichen mit der strukturangepassten Gruppe der Nichtpflegenden. Jede der erfassten psychosomatischen Beschwerden wird von Pflegenden häufiger berichtet als von Nichtpflegenden (Abb. 2). Allgemeine Müdigkeit wurde sowohl in der Gruppe der Pflegenden (55 %) als auch in der Gruppe der Nichtpflegenden (47 %) am häufigsten berichtet (Phi = 0,06). Körperliche Erschöpfung (Phi = 0,06), Kopfschmerzen (Phi = 0,06) und Schlafstörungen (Phi = 0,07) folgen mit einer Prävalenz von jeweils über 40 % in der Gruppe der Pflegenden dicht aufeinander. Auch bei Atemnot, einer im Vergleich zu den anderen Beschwerden weniger verbreiteten Beschwerde, zeigt sich ein Unterschied zwischen den Gruppen (Phi = 0,03). Bei den Muskel-Skelett-Beschwerden zeigt sich ein konsistentes Bild (Abb. 3). Nackenschmerzen wurden sowohl unter den Pflegenden (58 %) als auch unter den Nichtpflegenden (52 %) am häufigsten berichtet (Phi = 0,05). Darauf folgten die Rückenschmerzen (52 und 45 %; Phi = 0,05) und mit einer Prävalenz von jeweils weniger als 30 % in beiden Gruppen die weiteren Muskel-Skelett-Beschwerden. Alle Unterschiede zwischen den beiden Gruppen sind statistisch signifikant bei *p* < 0,01.

Des Weiteren ist die durchschnittliche Anzahl der gesundheitlichen Beschwerden unter den Pflegenden (M = 5,2; SD = 3,8) höher als in der Vergleichsgruppe der Nichtpflegenden (M = 4,2; SD = 3,6; SMD = −0,26; 95 %-KI [−0,32; −0,20]). Der Anteil der Personen ohne jegliche gesundheitliche Beschwerden ist unter den Pflegenden (13 %) niedriger als unter den Nichtpflegenden (19 %; SMD = 0,16; 95 %-KI [0,11; 0,22]). Diese Effekte sind statistisch signifikant bei *p* < 0,001.

### Effekte von Pflegeumfang und Arbeitszeit auf die Gesundheit

**Abb. 4:**
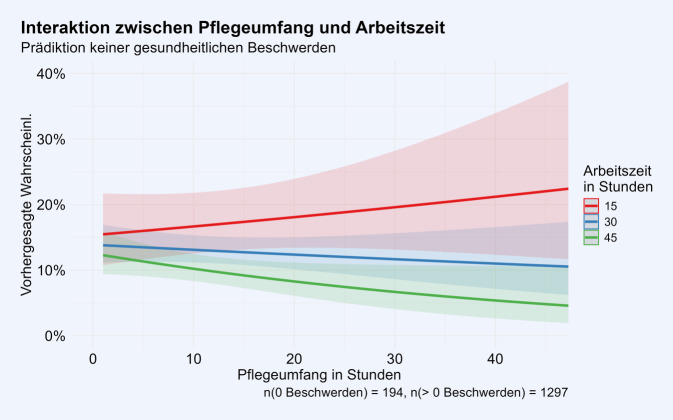
Interaktion zwischen Pflegeumfang und Arbeitszeit im logistischen Modell zur Vorhersage der gesundheitlichen Beschwerden als dichotome Variable (mindestens eine Beschwerde vs. keine Beschwerde)

Für die weitere Auswertung erfolgte die Fokussierung auf die Gruppe der pflegenden erwerbstätigen Personen mit dem Ziel herauszufinden, ob der Umfang der Pflege und der Erwerbsarbeit mit der Gesundheit im Zusammenhang stehen. Dafür haben wir ein logistisches Modell geschätzt, um die Abwesenheit von gesundheitlichen Beschwerden (i.e., eine „einwandfreie“ Gesundheit) vorherzusagen. Als Prädiktoren haben wir Alter, Geschlecht, Bildung, Pflegeumfang und Arbeitszeit sowie die Interaktion zwischen Pflegeumfang und Arbeitszeit in das Modell aufgenommen. Dabei zeigte sich ein statistisch signifikanter Interaktionseffekt zwischen Pflegeumfang und Arbeitszeit (*p* = 0,043). Die Interaktion ist in Abb. 4 dargestellt, wobei alle Kovariaten am Mittelwert konstant gehalten wurden. Der Effekt des Pflegeumfangs hängt von der Arbeitszeit ab. Bei einer Arbeitszeit von 15 h zeigt sich im Modell ein leicht positiver Zusammenhang zwischen Pflegeumfang und der Abwesenheit von gesundheitlichen Beschwerden, bei einer Arbeitszeit von 45 h ein leicht negativer Zusammenhang. Die detaillierten Ergebnisse der Regressionsanalyse sind tabellarisch im Zusatzmaterial online: Appendix 2 dargestellt.

## Diskussion

Ziel der Studie war es zu untersuchen, inwiefern die Pflegeverantwortung im privaten Umfeld bei Erwerbstätigen mit der Gesundheit im Zusammenhang steht. Im nächsten Schritt sollte analysiert werden, inwiefern innerhalb der Gruppe der Pflegenden der Umfang von sowohl der informellen Pflege als auch der Erwerbsarbeit mit der Gesundheit assoziiert ist.

Die Ergebnisse zeigen konsistent, dass pflegende Erwerbstätige im Durchschnitt einen schlechteren Gesundheitszustand aufweisen als Erwerbstätige ohne Pflegeverantwortung. Alle erfassten psychosomatischen und körperlichen Beschwerden traten ohne Ausnahme häufiger bei den Pflegenden auf. Außerdem war der Anteil der Personen ohne gesundheitliche Beschwerden unter den Pflegenden geringer als unter den Nichtpflegenden. Diese Befunde bestätigen die aktuelle Literatur [7, 8, 11] und erweitern sie um eine differenzierte Betrachtung spezifischer psychosomatischer und muskuloskeletaler Beschwerden.

Innerhalb der Gruppe der Pflegenden zeigte sich, dass der Effekt des Pflegeumfangs auf die Gesundheit von der Erwerbsarbeitszeit abhängt. Bei einer hohen Erwerbsarbeitszeit zeigte sich ein negativer Zusammenhang zwischen Pflegeumfang und Gesundheit. Dies bedeutet, dass Personen, die sowohl viel Care-Arbeit als auch Erwerbsarbeit ausüben, mit einer schlechteren Gesundheit in Verbindung stehen.

Die Frage, inwiefern der Umfang der Erwerbstätigkeit und der Pflege mit der Gesundheit informell pflegender Personen im Zusammenhang steht, wurde bereits auf Grundlage des Sozio-oekonomischen Panels (SOEP) untersucht [23]. Im SOEP werden sowohl der physische als auch der psychische Gesundheitszustand erfasst. Die Ergebnisse der Studie zeigten – kontraintuitiv – keinen statistisch signifikanten Effekt des Pflegeumfangs auf die physische Gesundheit [23]. Auch eine Interaktion zwischen Pflegeumfang und Arbeitszeit auf die physische Gesundheit erwies sich als statistisch nicht signifikant.

Für den psychischen Gesundheitszustand zeigte sich hingegen sehr wohl ein statistisch signifikanter und negativer Effekt des Pflegeumfangs. Unter Berücksichtigung der Interaktion von Pflegeumfang und Arbeitszeit wurde – im Einklang mit unseren Ergebnissen – deutlich, dass insbesondere die Kombination aus einem hohem Pflegeumfang und einer hohen Arbeitszeit mit einer schlechteren psychischen Gesundheit assoziiert ist.

Zusammenfassend sind die Ergebnisse hinsichtlich des psychischen Gesundheitszustands konsistent mit denen unserer Studie, jedoch nicht in Bezug auf den physischen Gesundheitszustand [23]. Eine mögliche Erklärung für diese unterschiedliche Befundlage könnte darin liegen, dass die Studie auf Basis des Sozio-oekonomischen Panels auch Personen umfasst, die nicht erwerbstätig sind, sowie solche, die nicht im privaten Umfeld pflegen, wodurch die Effekte innerhalb der Gruppe der pflegenden Erwerbstätigen womöglich unentdeckt blieben.

Die Untersuchung zeigt deutlich, dass pflegende Erwerbstätige vor besonderen Herausforderungen, die sich negativ auf ihre Gesundheit und ihr Wohlbefinden auswirken können, stehen. Angesichts dieser Ergebnisse wird die Notwendigkeit von Interventionen zur Gesundheitsförderung besonders evident [24]. Diese Interventionen sind wichtig, um die Gesundheit und das Wohlbefinden pflegender Erwerbstätiger nachhaltig zu verbessern [25, 26]. Dies kann indirekt sowohl zur Fachkräftesicherung als auch zur Aufrechterhaltung des Pflegearrangements beitragen. Entscheidend hierbei ist, dass die Bedürfnisse der pflegenden Erwerbstätigen bei der Entwicklung und Durchführung der Interventionen Beachtung finden [27, 28].

### Limitationen

Eine Limitation der Studie ist eine mögliche unbeobachtete Heterogenität zwischen der Gruppe der pflegenden Erwerbstätigen und der Vergleichsgruppe. Im Propensity Score Matching können grundsätzlich nur Kovariaten, die in der Befragung erfasst wurden, berücksichtigt werden. Es besteht dementsprechend die Möglichkeit, dass Unterschiede in den Zielgrößen, neben der Pflegeverantwortung, auch durch andere unbeobachtete Merkmale erklärt werden können.

Die Ergebnisse zur Gesundheit pflegender Angehöriger scheinen weitgehend unabhängig von der Operationalisierung von Gesundheit zu sein [29]. Es gibt aber Hinweise, dass die Befunde von der Operationalisierung der Pflegesituation und der Rahmenbedingungen abhängen. So gibt es deutliche Unterschiede, ob die pflegebedürftige Person im selben Haushalt lebt oder nicht [29]. Eine genauere Beschreibung der Pflegesituation war mit den in dieser Studie vorliegenden Daten nicht möglich.

Zudem erscheint es sinnvoll, interpersonale Variablen, wie etwa die Beziehungsqualität, in Studien zu berücksichtigen, da diese die Auswirkungen des Pflegeumfangs und der beruflichen Tätigkeit auf die Gesundheit möglicherweise moderieren können [30]. Weiterhin weisen Studien darauf hin, dass es Unterschiede zwischen pflegenden Männern und Frauen hinsichtlich psychosozialer Variablen und Bewältigungsstrategien gibt [11, 31]. Auch dieser Aspekt sollte in zukünftigen Forschungsarbeiten mit ausreichend großen Stichproben, die eine geschlechtsspezifische Differenzierung erlauben, untersucht werden.

## Fazit für die Praxis

- Pflegende Erwerbstätige sind eine Risikogruppe für gesundheitliche Beeinträchtigungen – sowohl bezüglich psychosomatischer als auch muskuloskeletaler Beschwerden.
- Der Gesundheitszustand pflegender erwerbstätiger Personen steht im Zusammenhang mit der Kombination aus Erwerbsarbeitszeit und Pflegeumfang.
- Eine Kombination aus hohem Erwerbsarbeitspensum und hohem Zeitaufwand für informelle Pflege ist mit einer schlechteren Gesundheit assoziiert.
- Das Pflegesystem in Deutschland steht schon heute unter Druck, und der demografische Wandel wird die Situation verschärfen. Um pflegende erwerbstätige Personen zu entlasten, sind Unterstützung durch Arbeitgeber und politische Maßnahmen unerlässlich.

## Supplementary Information

Appendix 1

Appendix 2

## Data Availability

Über das Forschungsdatenzentrum des Bundesinstituts für Berufsbildung ist ein Scientific-Use-File der BIBB/BAuA-Erwerbstätigenbefragung 2018 zu beziehen (https://www.bibb.de/de/120401.php).

## References

[1] Statistisches Bundesamt (2023) Pflegevorausberechnung: 1,8 Millionen mehr Pflegebedürftige bis zum Jahr 2055 zu erwarten. https://www.destatis.de/DE/Presse/Pressemitteilungen/2023/03/PD23_124_12.html. Zugegriffen: 16. Juli 2024

[2] Ribeiro O, Araújo L, Figueiredo D et al (2022) The caregiver support ratio in europe: estimating the future of potentially (un)available caregivers. Healthcare. 10.3390/healthcare10010011 35052175 10.3390/healthcare10010011 PMC8775661

[3] Klie T (2024) Pflegereport 2024. Die Baby-Boomer und die Zukunft der Pflege – Beruflich Pflegende im Fokus. https://caas.content.dak.de/caas/v1/media/64750/data/42a02e597e07646cc80c0ddbd1382a8f/dak-pflegereport-2024-ebook.pdf. Zugegriffen: 29. Okt. 2024

[4] Tesch-Römer C, Hagen C (2018) Ausgewählte Aspekte zur informellen häuslichen Pflege in Deutschland. https://www.dza.de/fileadmin/dza/Dokumente/Fact_Sheets/FactSheet_Inform_haeusl_Pflege.pdf. Zugegriffen: 29. Okt. 2024

[5] Lott Y, Hobler D, Pfahl S et al (2022) Stand der Gleichstellung von Frauen und Männern in Deutschland. Nr. 72. https://www.boeckler.de/fpdf/HBS-008259/p_wsi_report_72_2022.pdf. Zugegriffen: 15. Juli 2024

[6] Kuhlmey A, Budnick A (2023) Pflegende Angehörige in Deutschland: Vereinbarkeit von Pflege und Erwerbstätigkeit. Bundesgesundheitsblatt 66:550–556. 10.1007/s00103-023-03687-3 10.1007/s00103-023-03687-3 PMC10109225 37069275

[7] Büscher A, Peters L, Stelzig S et al (2023) VdK-Pflegestudie Abschlussbericht. Pflege zu Hause – zwischen Wunsch und Wirklichkeit. https://opus.hs-osnabrueck.de/frontdoor/deliver/index/docId/5236/file/VdK-Pflegestudie_Abschlussbericht_Februar_2023.pdf

[8] Janson P, Willeke K, Zaibert L et al (2022) Mortality, morbidity and health-related outcomes in informal caregivers compared to non-caregivers: a systematic review. Int J Environ Res Public Health. 10.3390/ijerph19105864 35627399 10.3390/ijerph19105864 PMC9141545

[9] Kaschowitz J, Brandt M (2017) Health effects of informal caregiving across Europe: a longitudinal approach. Soc Sci Med 173:72–80. 10.1016/j.socscimed.2016.11.036 27930918 10.1016/j.socscimed.2016.11.036

[10] Pinquart M, Sörensen S (2003) Differences between caregivers and noncaregivers in psychological health and physical health: a meta-analysis. Psychol Aging 18:250 12825775 10.1037/0882-7974.18.2.250

[11] Zwar L, König H‑H, Hajek A (2020) Psychosocial consequences of transitioning into informal caregiving in male and female caregivers: Findings from a population-based panel study. Soc Sci Med 264:113281. 10.1016/j.socscimed.2020.113281 32829215 10.1016/j.socscimed.2020.113281

[12] Pendergrass A, Weiß S, Rohleder N et al (2023) Validation of the Benefits of Being a Caregiver Scale (BBCS) – further development of an independent characteristic of informal caregiving. BMC Geriatr 23:26. 10.1186/s12877-022-03650-y 36641428 10.1186/s12877-022-03650-y PMC9840821

[13] Pysklywec A, Plante M, Auger C et al (2020) The positive effects of caring for family carers of older adults: a scoping review. Int J Care Caring 4:349–375. 10.1332/239788220X15925902138734

[14] Eggert S, Naumann D, Teubner C (2016) Vereinbarkeit von Beruf und Pflege: generelle und aktuelle Herausforderungen Betroffener. https://www.zqp.de/wp-content/uploads/Report_Vereinbarkeit_Beruf_Pflege_Pflegende_Angehoerige.pdf. Zugegriffen: 15. Juli 2024

[15] Pinquart M (2016) Belastungs- und Entlastungsfaktoren pflegender Angehöriger – die Bedeutung der Erwersbtätigkeit. https://www.zqp.de/wp-content/uploads/Report_Vereinbarkeit_Beruf_Pflege_Pflegende_Angehoerige.pdf. Zugegriffen: 11. Nov. 2024

[16] Hall A, Rohrbach-Schmidt D (2020) BIBB/BAuA-Erwerbstätigenbefragung 2018. BIBB-FDZ Daten- und Methodenbericht. https://www.bibb.de/dienst/publikationen/de/16401. Zugegriffen: 17. Juli 2024

[17] Hall A, Tiemann M, Siefer A et al (2018) BIBB/BAuA-Erwerbstätigenbefragung 2018. Erhebungsinstrument Fragebogenmaster für die CATI-Programmierung inkl. Variablenkennung. https://www.bibb.de/dokumente/pdf/a12_Fragebogen_ETB2018_Endfassung.pdf. Zugegriffen: 22. Juli 2024

[18] van Berk B, Ebner C, Rohrbach-Schmidt D (2023) Suchthaftes Arbeiten und Gesundheit: Empirische Befunde für Deutschland. https://www.boeckler.de/de/faust-detail.htm?sync_id=HBS-008589. Zugegriffen: 22. Juli 2024

[19] R Core Team (2024) R: a language and environment for statistical computing. R Foundation for Statistical Computing, Vienna

[20] Ho D, Imai K, King G et al (2011) Matchit: nonparametric preprocessing for parametric causal inference. J Stat Soft 42:1–28. 10.18637/jss.v042.i08

[21] Greifer N (2024) cobalt: covariate balance tables and plots. R package version 4.5.5. https://CRAN.R-project.org/package=cobalt. Zugegriffen: 22. Juli 2024

[22] Austin PC (2011) An introduction to propensity score methods for reducing the effects of confounding in observational studies. Multivariate Behavioral Res 46:399–424. 10.1080/00273171.2011.568786 10.1080/00273171.2011.568786 PMC3144483 21818162

[23] Bidenko K, Bohnet-Joschko S (2021) Vereinbarkeit von Beruf und Pflege: Wie wirkt sich Erwerbstätigkeit auf die Gesundheit pflegender Angehöriger aus? Gesundheitswesen 83:122–127. 10.1055/a-1173-8918 32645733 10.1055/a-1173-8918

[24] Janson P, Hung C‑W, Willeke K et al (2024) Wie wirksam sind nicht-pharmakologische Interventionen für pflegende Angehörige? Ein systematisches Review mit Metaanalysen. Gesundheitswesen. 10.1055/a-2340-1560 39146966 10.1055/a-2340-1560 PMC11849791

[25] Hetzel C, Bühne D, Michel W et al (2023) Mehr Freiraum für mich? Effekte einer Gruppenintervention für pflegende Angehörige anhand von Routinedaten der SVLFG-Pflegekasse. Z Gerontol Geriat 56:477–483. 10.1007/s00391-022-02086-8 10.1007/s00391-022-02086-8 35852589

[26] Hetzel C, Schaller J, Michel W et al (2024) Effekte eines einwöchig-stationären Gesundheitsprogramms für pflegende Angehörige gemeinsam mit ihrer pflegebedürftigen Person (Pflege-Tandem der SVLFG) – eine quasiexperimentelle Studie. Gesundheitswesen. 10.1055/a-2305-0146 38604607 10.1055/a-2305-0146 PMC11849786

[27] Hampel S (2020) Gesundheitsvorstellungen und Gesundheitshandeln pflegender Angehöriger von Menschen mit Demenz. Z Gerontol Geriat 53:29–34. 10.1007/s00391-019-01665-6 10.1007/s00391-019-01665-6 31820088

[28] Lindt N, van Berkel J, Mulder BC (2020) Determinants of overburdening among informal carers: a systematic review. BMC Geriatr 20:304. 10.1186/s12877-020-01708-3 32847493 10.1186/s12877-020-01708-3 PMC7448315

[29] Kaschowitz J, Lazarevic P (2020) Bedeutung des Gesundheitsindikators bei der Analyse der Gesundheitsfolgen informeller Pflege: Anderer Indikator, anderes Ergebnis? Z Gerontol Geriat 53:10–16. 10.1007/s00391-019-01663-8 10.1007/s00391-019-01663-8 31802211

[30] Naef R, Hediger H, Imhof L et al. (2017) Variances in family carers’ quality of life based on selected relationship and caregiving indicators: A quantitative secondary analysis. Int J Older People Nurs 12:e12138. https://doi.org/10.1111/opn.12138 10.1111/opn.12138 27863032

[31] Geyer J (2016) Informell Pflegende in der deutschen Erwerbsbevölkerung: Soziodemografie, Pflegesituation und Erwerbsverhalten. ZQP-Themenreport: Vereinbarkeit von Beruf und Pflege. Zentrum für Qualität in der Pflege, S 24–43

