# Supplementary material for: Assessing the health of working informal caregivers: Analysis of an employment survey using propensity score matching
Source: Z Gerontol Geriatr. 2025 Jan 6;58(4):296–302. [Article in German] doi: 10.1007/s00391-024-02387-0 (PMC12238193; doi:10.1007/s00391-024-02387-0)
Supplement: Supplementary file 1 — Appendix 1 [file 391_2024_2387_MOESM1_ESM.docx]

# Appendix 1

Die Tabelle in Appendix 1 zeigt eine Beschreibung der Stichprobe vor dem Matching, differenziert nach Pflegeverantwortung. Alle im Modell des Propensity Score Matching berücksichtigten Kovariaten sind aufgelistet. Personen mit fehlenden Werten bei einer dieser Kovariaten wurden ausgeschlossen, da diese nicht im Propensity Score Matching berücksichtigt werden können.

Tabelle: Deskription der Kovariaten des Propensity Score Matching (vor dem Matching).

| **Merkmal** | **Pflegende Erwerbstätige**  n = 1.505 | **Nicht-Pflegende Erwerbstätige**  n = 17.822 |
| --- | --- | --- |
| **Alter** | 50,9 (9,7) | 46,9 (11,4) |
| **Geschlecht** |  |  |
| männlich | 38,3 % | 51,5 % |
| weiblich | 61,7 % | 48,5 % |
| **Familienstand** |  |  |
| verheiratet | 63,2 % | 55,2 % |
| ledig | 20,9 % | 29,5 % |
| geschieden | 13,0 % | 12,5 % |
| verwitwet | 3,0 % | 2,7 % |
| **Kinder** |  |  |
| ja | 77,0 % | 67,0 % |
| nein | 23,0 % | 33,0 % |
| **Kinder unter 18 Jahre im Haushalt** |  |  |
| ja | 27,6 % | 32,7 % |
| nein | 72,4 % | 67,3 % |
| **Bildung** |  |  |
| Ohne Berufsabschluss | 4,9 % | 5,2 % |
| Berufsausbildung | 55,0 % | 47,7 % |
| Aufstiegsfortbildung | 8,7 % | 8,0 % |
| Hochschulabschluss | 31,4 % | 39,1 % |
| **Branche** |  |  |
| Öffentlicher Dienst | 31,5 % | 27,4 % |
| Industrie | 14,8 % | 17,9 % |
| Handwerk | 5,8 % | 7,8 % |
| Handel | 8,2 % | 8,8 % |
| Sonstige Dienstleistung | 26,9 % | 28,3 % |
| anderer Wirtschaftsbereich | 7,0 % | 6,0 % |
| Gewerkschaften, Verbände | 5,8 % | 3,8 % |
| **Jahre der Betriebszugehörigkeit** | 16,8 (12,4) | 14,0 (11,3) |
| **Arbeitslosigkeit** |  |  |
| keine Arbeitslosigkeit | 60,4 % | 63,7 % |
| weniger als ein halbes Jahr | 13,0 % | 12,9 % |
| 1 Jahr | 14,7 % | 13,9 % |
| 2 Jahre | 5,8 % | 4,8 % |
| Länger als 2 Jahre | 6,2 % | 4,7 % |
| **Nebentätigkeit** |  |  |
| keine Nebentätigkeit | 88,9 % | 90,9 % |
| mindestens eine Nebentätigkeit | 11,1 % | 9,1 % |

^1^Mittelwert (SD) / %
