# Supplementary material for: Assessing the health of working informal caregivers: Analysis of an employment survey using propensity score matching
Source: Z Gerontol Geriatr. 2025 Jan 6;58(4):296–302. [Article in German] doi: 10.1007/s00391-024-02387-0 (PMC12238193; doi:10.1007/s00391-024-02387-0)
Supplement: Supplementary file 2 — Appendix 2 [file 391_2024_2387_MOESM2_ESM.docx]

# Appendix 2

Logistisches Modell zur Vorhersage keiner gesundheitlicher Beschwerden
(Nagelkerke's Pseudo-R^2^ = 0,046).

| **Merkmal** | **N** | **OR** | **95% CI** | **p-Wert** |
| --- | --- | --- | --- | --- |
| Alter | 1,378 | 1.00 | 0.99, 1.02 | 0.7 |
| Geschlecht |  |  |  |  |
| männlich | 535 | — | — |  |
| weiblich | 843 | 0.46 | 0.32, 0.65 | <0.001 |
| Bildung |  |  |  |  |
| Ohne Berufsabschluss | 67 | — | — |  |
| Berufsausbildung | 748 | 1.39 | 0.61, 3.74 | 0.5 |
| Aufstiegsfortbildung | 121 | 2.01 | 0.77, 5.93 | 0.2 |
| Hochschulabschluss | 442 | 1.96 | 0.85, 5.37 | 0.15 |
| Pflegeumfang in 10 Stunden | 1,378 | 1.30 | 0.90, 1.83 | 0.13 |
| Arbeitszeit in 10 Stunden | 1,378 | 0.92 | 0.77, 1.11 | 0.4 |
| Pflegeumfang * Arbeitszeit | 1,378 | 0.90 | 0.80, 1.00 | 0.043 |
